# Views and perspectives on the indoleamines serotonin and melatonin in plants: past, present and future

**DOI:** 10.1080/15592324.2024.2366545

**Published:** 2024-06-20

**Authors:** Lauren A.E. Erland

**Affiliations:** Agriculture, University of the Fraser Valley, Chilliwack, BC, Canada

**Keywords:** melatonin, serotonin, indoleamine, phytohormone, antioxidant, stress, plant physiology

## Abstract

In the decades since their discovery in plants in the mid-to-late 1900s, melatonin (*N*-acetyl-5-methoxytryptamine) and serotonin (5-methoxytryptamine) have been established as their own class of phytohormone and have become popular targets for examination and study as stress ameliorating compounds. The indoleamines play roles across the plant life cycle from reproduction to morphogenesis and plant environmental perception. There is growing interest in harnessing the power of these plant neurotransmitters in applied and agricultural settings, particularly as we face increasingly volatile climates for food production; however, there is still a lot to learn about the mechanisms of indoleamine action in plants. A recent explosion of interest in these compounds has led to exponential growth in the field of melatonin research in particular. This concept paper aims to summarize the current status of indoleamine research and highlight some emerging trends.

## 1. Introduction

Melatonin (*N*-acetyl-5-methoxytryptamine) and serotonin (5-methoxytryptamine) are indoleamine hormones that are produced across all Kingdoms of life on Earth. They have been implicated in the control of almost every aspect of plant life from reproduction to morphogenesis, plant environmental perception and stress responses^1^. The two indoleamines, and melatonin in particular, were originally proposed to function as minor auxins in plants. While their interaction with auxin has now been established, with activity in some cases being auxin-dependent,^2^ so too has their status as a distinct class of plant hormone and plant growth regulator.^3,4^ Melatonin and serotonin function both through direct action, as well as modification of transcriptional pathways and initiation of downstream signaling cascades, most notably calcium, nitric oxide and reactive oxygen species signaling pathways.^5^ Both are still better recognized for their role in the mammalian nervous, gastrointestinal and reproductive systems, but in recent decades, they have been labeled as pleiotropic hormones, master regulators and mediators of stress in plants, with the potential to help maintain agricultural productivity under future climates.^6–8^

**Figure 1.**
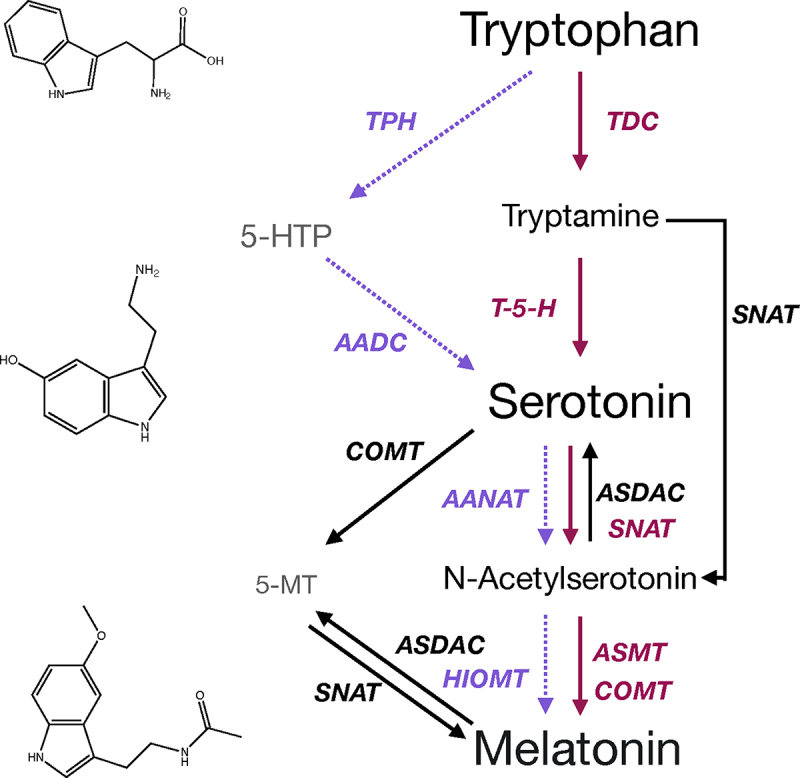
Overview of the indoleamine biosynthetic pathway in plants as compared to animals. The primary biosynthetic pathway in animals is represented by dashed purple arrows, the primary biosynthetic pathway in plants in solid pink arrows and alternate pathways described to date in plants in solid black arrows. Enzyme names are colored to match the associated arrows. AADAC, aromatic amino acid deacetylase; AANAT, aromatic amino acid N-acetyltransferase, ASDAC, acetylserotonin-deacetylase ASMT, acetylserotonin-O-methyltransferase; COMT, caffeic acid-O-methyltransferase; HIOMT, hydroxyindole-O-methyltransferase; SNAT, serotonin-N-acetyltransferase; T-5-H, tryptamine-5-hydroxlyase; TDC, tryptophan decarboxylase; TPH, tryptophan hydroxylase.

Melatonin and serotonin are synthesized from the aromatic amino acid tryptophan. In plants, the primary pathway of biosynthesis proceeds via first carboxylation to form tryptamine by tryptophan decarboxylase (TDC),^9,10^ followed by hydroxylation by tryptamine-5-hydroxylase (*T*-5-H).^11^ Unlike in the animal system where the hydroxylation is the more highly regulated reaction, in plants, TDC is the more highly regulated of the two steps, with the conversion by *T*-5-H occurring rapidly and with seemingly few controls.^4^ Surprisingly, TDC is actually a rather nonspecific enzyme, first identified as an aromatic amino acid decarboxylase, which is able to decarboxylate several aromatic amino acids.^12,13^ This low specificity serves a role in animals to allow for broader functionality of the enzyme due to the restriction of dietary tryptophan sources, as it is an essential amino acid not produced by animals; however, it is unclear why this lack of specificity may exist in the plant system. This highlights the possibility of as yet undescribed regulatory mechanisms. Additionally, there is some evidence for the presence of an alternate biosynthetic pathway for serotonin in plants, via the mammalian biosynthetic intermediate 5-hydroxytryptophan (5-HTP), which is produced via hydroxylation of tryptophan,^14^ or via 5-hydroxylation of the phytohormone indole-3-acetic acid (IAA) or auxin.^15^ The enzymes that may catalyze these reactions in plants have not yet been identified; however, 5-HTP has been quantified in several plant species and has been shown to be produced from IAA in an enzyme preparation from *Sedum morganianum* E. Walther indicating that the pathway is present.^15^ Serotonin is converted to melatonin via the intermediate *N*-acetylserotonin,^16,17^ via the action of first serotonin *N*-acetyl transferase (SNAT), to form *N*-acetylserotonin, which is then converted by either *N*-acetylserotonin methyltransferase (ASMT) or caffeic acid *O*-methyltransferase (COMT), to melatonin in the primary pathway.^18,19^ Diverse alternate pathways for biosynthesis have been identified. For example, SNAT may produce melatonin without going through serotonin as an intermediate using tryptamine as a substrate,^20^ and 5-methoxytryptamine (5-MT) may be used as an intermediate in lieu of NAS.^21^ Reverse carbon flow through the pathway has also been described, making the indoleamine pathway highly redundant and complex, and the potential implication of these alternate routes on physiological function is an open area of research and speculation (Figure 1).

**Box 1. Core Hypotheses in Plant Indoleamine Research**

1. Melatonin and serotonin are ancient molecules which evolved in the first prokaryotic forms of life as antioxidants which enabled survival under increasingly oxygenated atmospheres.
2. Indoleamines function as a pathway rather than individual molecules to achieve plant growth outcomes.
3. Melatonin and serotonin are plant hormones which have dual stress defense and morphogenetic functions in plants, enabling plant perception and response to (changing) environments.
4. Indoleamines mechanism of action is analogous to that of other plant signaling molecules. Effects are modulated through interactions with a receptor which induces transcriptional regulation and downstream pathways in both its morphogenetic and stress defense functions.
5. Indoleamines can function non-specifically as direct antioxidants in response to stress.

## 2. Current and Emerging Trends in Indoleamine Research

In our 2021 review, we identified several key trends in melatonin research including abiotic stress responses, root morphogenesis, response to light, interkingdom communication, protection of reproductive tissues and signaling cascades.^1^ These trends continue to be active areas of research. Using the same search strategy used in that systematic review, but for the time period from 2021 to 2024, we find that the size of the overall literature on melatonin in plants alone has almost doubled in size. From 1995 to 2021, 693 research articles were published on the topic of melatonin AND plants (Web of Science). From 2021 to 2024, an additional 823 original research papers were published (WoS query link: https://www.webofscience.com/wos/woscc/summary/6e6df7c7-cb17-4b17-a54c-31ae82e056d6-cc32b66c/relevance/1; Supplementary File 1). In contrast, research in the area of serotonin, and other neurohormones including catecholamines, has been much more moderate with a search for serotonin AND plants with no date restrictions resulting in 397 published articles after manual curation (WoS Query: https://www.webofscience.com/wos/woscc/summary/646a9f72-71b9-46cb-b45a-236fc717ec3b-cc33a1d7/relevance/1; Supplementary File 2).

**Figure 2.**
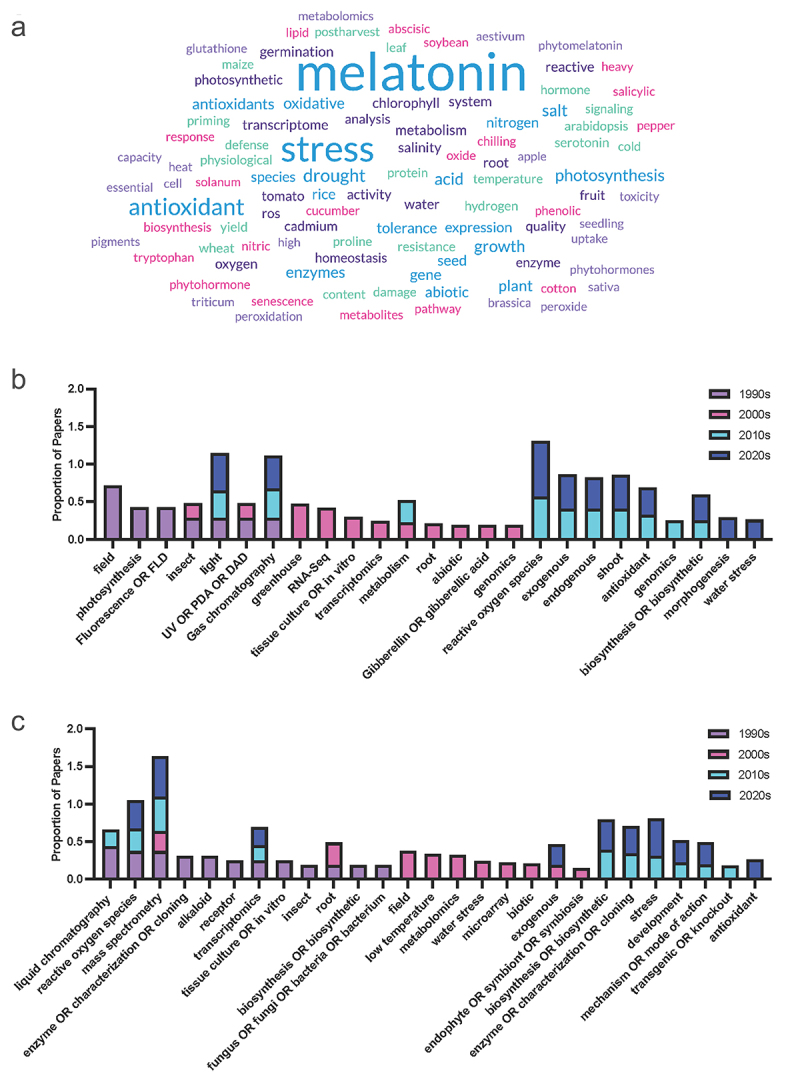
(a) Word cloud representing the most commonly included author keywords for original research articles included melatonin and plants published from 2021 to 2024. Word cloud was created in www.freewordcloudgenerator.com. (b) Top query terms by decade for melatonin and (c) Top query terms by decade for serotonin.

A search of the proportion of the query terms (Supplementary File 2) across the literature grouped by decade for the last 30 years was used to identify increasing and decreasing trends in the literature, which were compared between melatonin and serotonin (Supplementary File 3 & 4). To account for the exponential growth in melatonin over this time period, queries were normalized to a total number of papers published each year to generate a proportion of articles mentioning the term in their title or abstract in each decade. While serotonin has a longer history with its discovery predating that of melatonin in plants by 40 years, a comparison from the 90s forward only was used to align with the discovery of melatonin in plants in 1995 (foods)^22,23^ and 1997 (growing plants).^24^ Some clear trends were identified in these results and were used to identify the trends summarized in the following sections, with an overview of these trends over time provided in Figure 2.

### 2.1. Global distribution of researchers is distinct between melatonin and serotonin research

**Figure 3.**
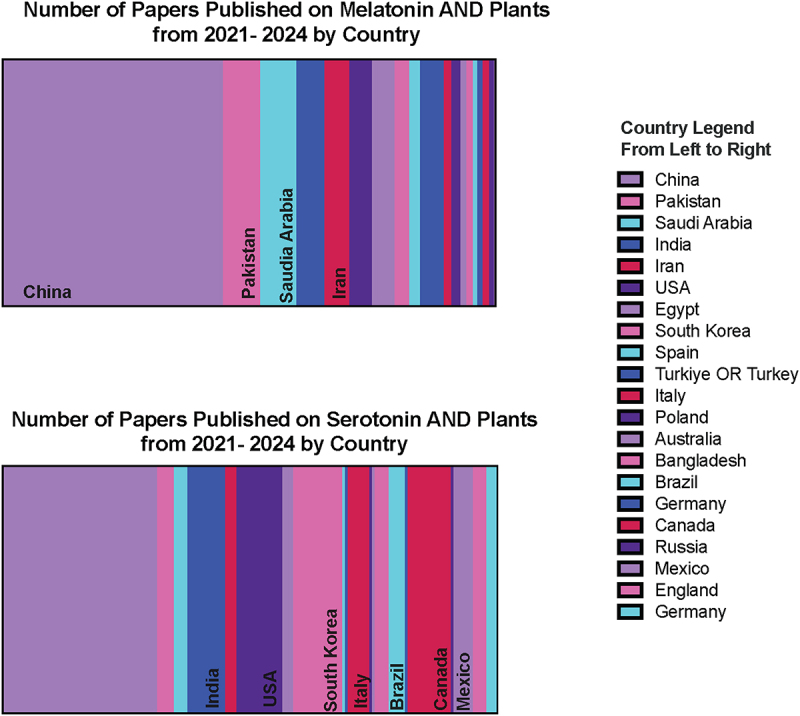
Distribution of country of publication of articles published on melatonin and plants (top) or serotonin and plants according to a Web of Science search between 2021 and 2024. Only countries which included more than 2% of total papers published in the query are included.

The People’s Republic of China represented the largest publisher of these articles for both melatonin and serotonin; however, distribution of research efforts globally beyond this varied between melatonin and serotonin with Europe and the Americas publishing a greater proportion of the papers on serotonin, while the Middle East and Asia seem to have a greater focus or contribution to the melatonin literature (Figure 3). These results were not altogether unexpected; it is of note that while the two hormones are closely related biochemically, there is distinct interest and research groups focusing on the two separately.

### 2.2. Species studied continue to focus on commercially, scientifically and agriculturally relevant species

**Figure 4.**
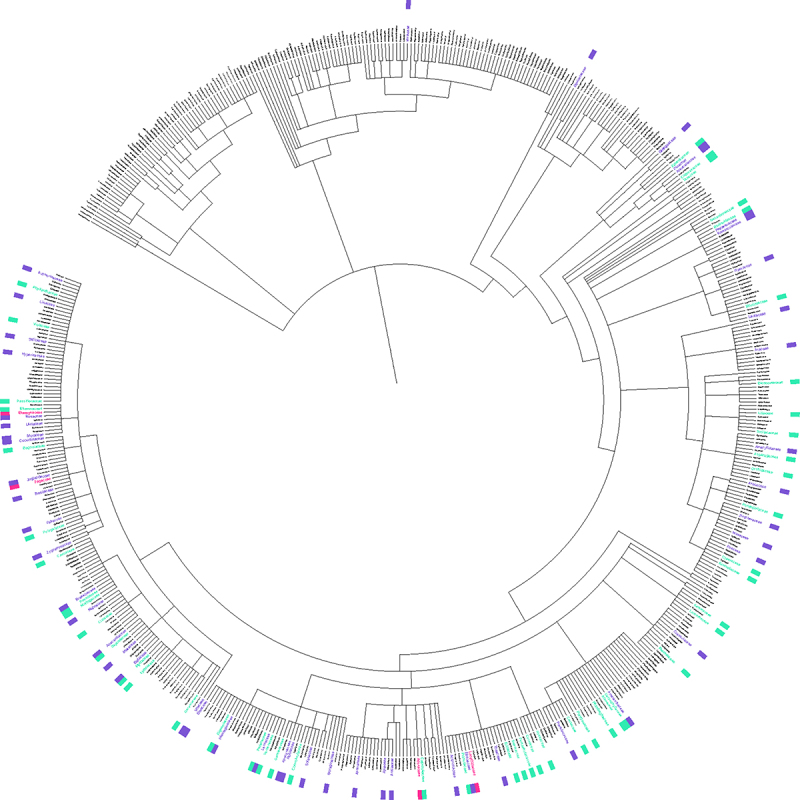
Phylogenetic tree of species in which melatonin (blue), serotonin (pink) or both (purple) have been studied and mapped to all plant families. Tree was constructed using PhyloT v2 and visualized in iTOL using the annotation spreadsheet editor. A list of all plant families was acquired from World Flora Online,^26,27^ family and species names were confirmed using the taxonomic name resolver service.^28^ An interactive version of this figure can be viewed at https://itol.Embl.de/export/5067182177413091708903228.

**Figure 5.**
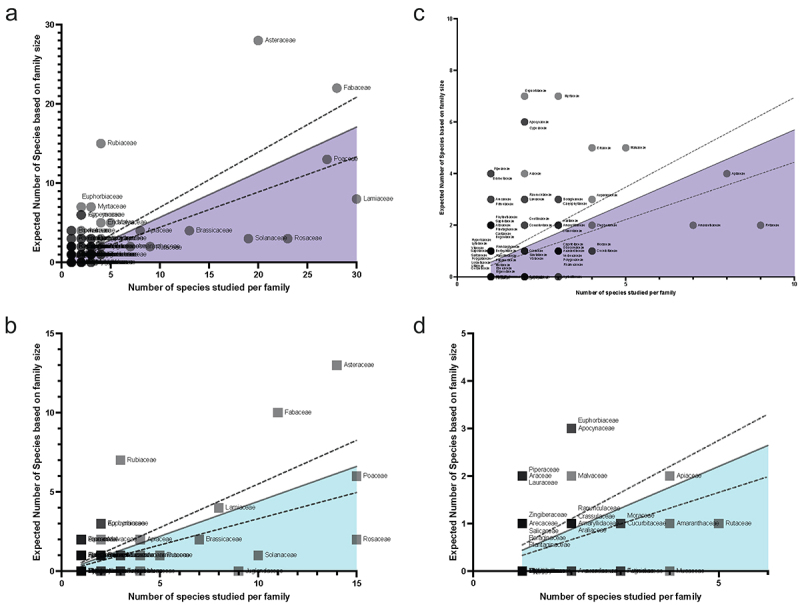
Distribution of species studied in melatonin (a & c) and serotonin (b & d) by family. The line of best fit is the linear regression line of the actual number of species studied by family and the predicted number of species that would be studied if studies were proportional to the total number of species.^29^ Dotted lines represent the 95% confidence interval. Families below the line (shaded portion) are underrepresented in the field, while those above the line are overrepresented. Panels A & B show the full plots, while c & d provide a zoomed-in view of the plots to the left. Individual points are transparent, with darker circles or squares representing a greater number of families at a given coordinate.

In a review of the species described in the literature starting from previously compiled species lists and querying the results of the Web of Science Searches described above, melatonin and serotonin were found to have been investigated in 98, or about 15% of plant families and > 390 species. In spite of this diversity, the work that has been done has overwhelmingly focused on angiosperms (Figure 4) and even more narrowly a few economically valuable families, as well as model species such as *Arabidopsis* (Supplementary File 3 & 4). The top families studied for melatonin including Poaceae (396 papers), Solanaceae (*n* = 347), Brassicaceae (*n* = 259), Rosaceae (*n* = 250), Fabaceae (*n* = 136), Cucurbitaceae (*n* = 80) and Lamiaceae (*n* = 61). To examine the overall diversity and representation of species studied, we used a previously described approach^25^ to identify families overrepresented and underrepresented by comparing the total number of species investigated in either body of the literature for each family, with predicted number of species that would be expected to have been studied if it was proportional to estimated family size (Figure 5). For both melatonin and serotonin, the Asteraceae, Fabaceae and Rubiaceae had the greatest relative species diversity investigated, while the Solanaceae, Brassicaceae and Rosaceae were all under-investigated in both indolamines, the Lamiaceae in the melatonin literature and Juglandaceae in the serotonin literature. The serotonin literature had a large diversity of species which are associated with their psychoactive effects due to the interactions of, for example, neuroactive alkaloids with serotonin receptors in the mammalian nervous system. Considering the relative size of the fields, it is interesting to note the relatively high diversity of species studied in the field of serotonin research compared to melatonin.

### 2.3. Advances in mass spectrometry are leading to a shift in quantification approaches

**Figure 6.**
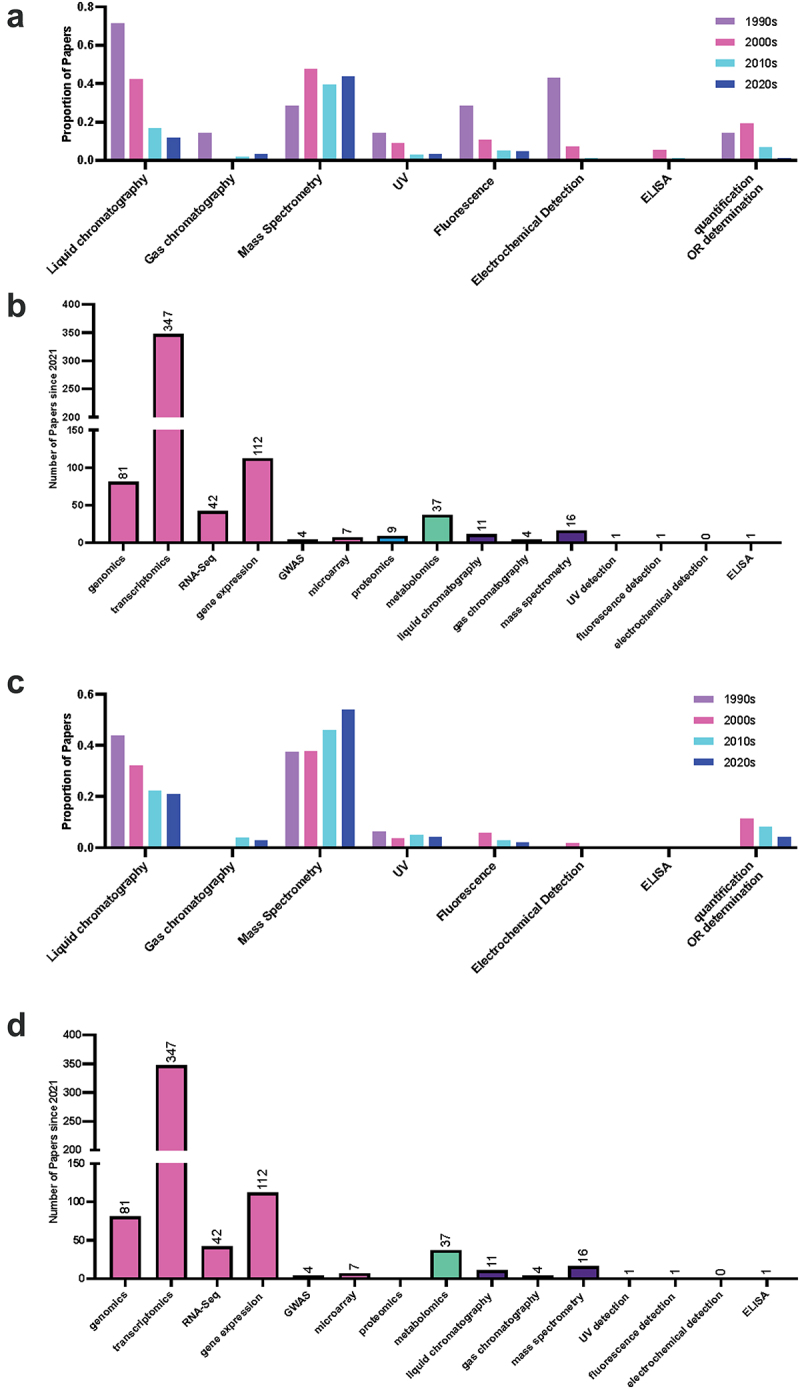
Proportion of publications per decade mentioning quantification query terms in the abstract of title for (a) melatonin and (c) serotonin literature and the number of publications using determination approaches since 2021 for (b) melatonin and (d) serotonin. Query terms used were: UPLC or HPLC or liquid chromatography or liquid-chromatography, GC or gas chromatography or gas-chromatography, MS or mass spectrometry or mass-spectrometry, UV or PDA or DAD, Fluorescence Detect or FLD, ECD or electrochemical detect, ELISA or enzyme-linked immunosorbent assay or EIA or enzyme immunoassay, gene expression or qPCR or RT-PCR, GWAS (genome-wide association studies).

The largest class of query terms showing a change in trend was quantification methods, with the term quantification itself showing a decreasing trend in recent decades for melatonin (Figure 6, Table 1). As a broad term, this represents the initial excitement and flurry of literature describing the presence of melatonin in new species following its discovery. The shift in the types of quantification methods reported in the literature is unsurprising, as early methods for detection relied heavily on techniques such as enzyme-linked immunosorbent assay (ELISA) and electrochemical detection (ECD), followed by ultraviolet (UV) and fluorescence detection (FLD) toward mass spectrometry (MS). Several papers do not reference a detection method in the abstract or title, despite specifying a separation method (usually via liquid chromatography; LC); these papers generally tend to have used UV detection, when the method is further investigated in the paper, suggesting that UV detection is likely underrepresented. This represents a broader challenge in assessing the quality of the work, as while this may be a shorthand usage, it could also signal a lack of familiarity by some authors with the approach. Recent advances in technology and reduction in costs in MS have likely enabled this shift toward MS-based methods for quantification of both hormones. MS-based approaches represent a new opportunity in indoleamine research, due to their sensitivity and specificity, enabling quantification in a greater diversity of tissues and species at a larger scale, while also representing new challenges for the field. We have previously described challenges with melatonin stability during extraction and the diversity of quantification methods published in the literature.^26^ Plant hormones are in part defined by their location, with the classical definition paraphrased from Went’s (1937) definition of hormones as compounds present at low concentrations which are transported to a location where they have an effect.^27^ Interest in localization of the enzymes responsible for biosynthesis is well established, and papers including queries for localization show an increasing trend for both melatonin and serotonin. New advances including MS imaging which enables spatial localization of chemicals are likely to further expand our understanding of plant hormones. Currently, most instruments and methods lack adequate sensitivity to accurately and reproducibly detect very low abundance molecules, including plant hormones, at adequate spatial resolution to be able to be useful for biological inference. New approaches and technology are already emerging using desorption electrospray ionization (DESI) MS which can quantify the indole phytohormone auxin at a resolution of 200 µm.^28^ As we lack reporter genes for both melatonin and serotonin, this will offer an *in situ* alternative to quantum-dot imaging^29^ or histology-based^30^ approaches currently available to study the localization of the indoleamines. This is likely to lead to new and exciting discoveries in the mechanism and function of indoleamines in plants. After the discovery of melatonin and serotonin in plants, the initial research enthusiasm centered around the search for equivalent functions and mechanisms for these hormones in animals and plants. This included a focus on the identification of receptors and the modulation of biological rhythms in particular. Since these earlier days, research interest in melatonin as a controller of circadian rhythms has dropped off, with both the total number of papers and the proportion of papers dropping off by the 2020s.

**Table 1.** Summary of indoleamine quantification methodologies by plant species since 2021.

Plant Species	Common Name	MEL	5HT	LC	GC	MS	PDA/ UV	FL	ECD	ELISA	Reference
*Brassica juncea*	**Mustard**	**X**		**X**			**X**				^112^
*Musa sapientum*	**Banana**	**X**	**X**	**X**		**X**		**X**			^113^
*Mangifera indica*	**Mango**	**X**	**X**	**X**		**X**		**X**			^113,114^
*Carica papaya*	**Papaya**	**X**		**X**				**X**			^113^
*Antidesma thwaiteaianum*	**Makmao**	**X**						**X**			^113^
*Ananas comosus*	**Pineapple**	**X**	**X**	**X**		**X**		**X**			^113,114^
*Morus alba*	**Mulberry**	**X**	**X**	**X**		**X**					^114^
*Solanum lycopersicum* var. cerasiforme	**Tomato**	**X**	**X**	**X**		**X**					^114^
*Solanum lycopersicum cv. Moneymaker*	**Tomato**		**X**	**X**		**X**					^115^
*Psidium guajava*	**Guava**	**X**	**X**	**X**		**X**					^114^
*Tamarindus indica*	**Tamarind**	**X**	**X**	**X**		**X**					^114^
*Syzygium samarangense*	**Rose apple**	**X**	**X**	**X**		**X**					^114^
*Citrullus lanatus*	**Watermelon**	**X**	**X**	**X**		**X**					^114^
*Citrus sinensis*	**Orange**	**X**	**X**	**X**		**X**					^114^
*Citrus reticulata*	**Orange**	**X**		**X**				**X**			^113^
*Dimocarpus longan*	**Longan**	**X**	**X**	**X**		**X**					^114^
*Cucumis melo var. cantalupo*	**Cantaloupe**	**X**	**X**	**X**		**X**					^114^
*Passiflora edulis*	**Red passion fruit**	**X**	**X**	**X**		**X**					^114^
*Passiflora edulis*	**Yellow passion fruit**	**X**	**X**	**X**		**X**					^114^
*Ziziphus jujuba*	**Jujube**	**X**	**X**	**X**		**X**					^114^
*Gossypium hirsutum*	**Cotton**	**X**		**X**		**x**					^116^
*Wollemia nobilis*	**Wollemi pine**	**X**		**X**		**x**					^117^
*Araucaria heterophylla*	**Norfolk Island Pine**	**X**		**X**		**X**					^117^
*Cornus mas*	**Cornelian cherry**	**X**		**X**				**X**			^118^
*Tilia tomentosa*	**Linden**	**X**	**X**	X*			**X**				^119^
*Foeniculum vulgare*	**Fennel**	**X**	**X**	X*			**X**				^119^
*Salvia officinalis*	**Sage**	**X**	**X**	X*			**X**				^119^
*Matricaria chamomilla*	**Chamomile**	**X**	**X**	X*			**X**				^119^
*Solanum lycopersicum*	**Tomato**	**X**		**X**		**X**					^120^
*Dracocephalum kotsychi*		**X**		X*			**X**				^121^
*Capsicum annuum*	**Sweet Pepper**	**X**		**X**		**X**					^122^
*Triticum aestivum*	**Wheat**	**X**		**X**		**X**	**X**				^123^
*Oryzae sativa*	**Rice**	**X**	**X**	**X**	**X**	**X**					^124,125^
*Suaede spp.*		**X**			**X**	**X**					^126^
*Solanum tuberosum*	**Potato**	**X**								**X**	^127^
*Prunus salicina*	**Angeleno plum**	**X**		**X**		**X**	**X**				^128^
*Spirodela polyrhiza*	**Duckweed**		**X**		**X**	**X**					^129^
*Helianthus annuus*	Sunflower**		**X**	**X**		**X**					^130^
*Hippophae rhamnoides*	**Sea Buckthorne**		**X**	**X**		**X**					^131^
*Elatostema papillosum*			**X**		**X**	**X**					^132^

*Paper specifies only ‘quantification by HPLC’ in the abstract, with no detection method.

**Wrong reference to method paper referenced.

Indoleamine research has also seen an uptick in the adoption of other novel technologies including a step growth, particularly from 2000 to 2010, in uptake of -OMICS strategies starting with transcriptomics, followed by genomics, and then metabolomics further demonstrating the impact of new technologies on the field (Figure 2). Gene and transcript centered -OMICS and determination strategies remain the most popular discovery and determination approaches with transcriptomics, gene expression (e.g. via RT-qPCR), genomics and RNA-Seq being at a frequency orders of magnitude higher than quantification strategies such as MS or other downstream -OMICS approaches, including proteomics and metabolomics. This is likely due in part to the lower cost barrier to entry, for example, of RT-qPCR studies, as well as greater comfort and expertise on the part of biologists working in these systems (Figure 6b and d). It will be exciting to see updates in the field as more multi-omics studies are published in this area which are able to link gene expression to final (chemical) phenotype.

### 2.4. Expansion in interest in interactions between the indoleamine biosynthetic pathway, metabolites and other plant signaling and secondary metabolite pathways

**Figure 7.**
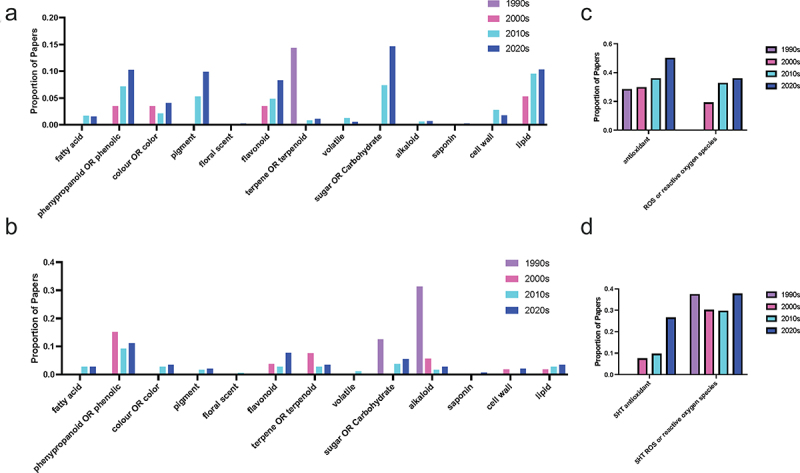
Proportion of publications per decade with mentions by chemical class for (a, c) melatonin and (b, d) serotonin literature.

All plant hormones function through interactions with other biochemical pathways and signaling networks; in particular, connections between melatonin, serotonin and the phenylpropanoid pathway are notably active areas of research (Figure 7). Melatonin and serotonin have been found to function synergistically with the phenylpropanoid pathway in the bryophyte moss *Plagiomnium cuspidatum* (Hedw.) T.J. Kop.^31^ Melatonin has been found to be able to modify floral scent in ginger lily (*Hedychium coronarium* J König) where both monoterpene and phenylpropanoid contents were increased in response to melatonin treatment. Serotonin-phenolic conjugates including feruloyl- and coumaroyl-serotonin are well-known stress-responsive compounds produced in association with damage to the plant cell wall, particularly through insect feeding and pathogen challenge. Caffeic acid *O*-methyltransferase is an *O*-methyltransferase enzyme which is involved in both lignin and melatonin biosynthesis. In the melatonin biosynthetic pathway, COMT can mediate the conversion of NAS to melatonin.^18,32^ Recent work using phylogenetic approaches has proposed that the COMT gene is evolutionarily more ancient than ASMT which is used both in plants and other species to catalyze this same reaction^33,34^ and that ASMT evolved from COMT.^35^ As our understanding of the melatonin biosynthetic pathway continues to evolve, the interactions between melatonin and the phenylpropanoid pathway, particularly flavonoids, to which caffeic acid belongs, as well as the terms ‘colo(u)r’ and ‘pigments’, continue to expand. This has been associated with a reduction in interest in interactions between the indoleamines and other classes of secondary metabolites including the terpenes and the alkaloids. Interest in serotonin and alkaloids was driven in earlier decades by the mechanism of many alkaloids on serotonin receptors and reuptake inhibitors in human nervous systems, and particularly the potential to use pharmaceuticals as agonists and antagonists in the search for plant indoleamine receptors and mechanisms of action.^36,37^

An interesting area of interaction between chemical classes is between melatonin, serotonin and fatty acids. Melatonin has been found to modify membrane fatty acid composition, maintain membrane fluidity under temperature stress, and protect both cellular, chloroplast and mitchondrial membranes from lipid peroxidation and damage, primarily in the context of enhanced stress tolerance and improved photosynthetic capacity.^38^ Serotonin has also been found to regulate phosphatidyl inositol signaling in response to red light treatment, downstream of calcium signaling in maize protoplasts.^39–42^ Little work has been done to investigate the potential influence melatonin may be able to exert on fatty acid signaling; however, some of our recent research has pointed toward a potential interaction between melatonin, fatty acids, flavonoids and rhomboid receptor proteins.^36^

Increases in interest in melatonin and sugars or carbohydrates (Figure 7) may in part be explained by the increase in studies examining the effects of melatonin on photosynthetic efficiency under stress (Figure 7). While the capacity for melatonin to improve photosynthesis and downstream carbon fixation reactions has an obvious benefit for the agricultural production of starch crops in increasingly stressful global climates,^43^ melatonin also has the capacity to modify another important function of sugars in plants: sucrose signaling and transport.^44^ Sucrose signaling in plants is known to interact with other hormone pathways leading to diverse effects on plant growth.^45^ Very little information is available for either melatonin or serotonin in this area, representing another under-investigated area in plant indoleamine research.

### 2.5. Shift from understanding physiological functions toward stress tolerance and climate resilience

**Figure 8.**
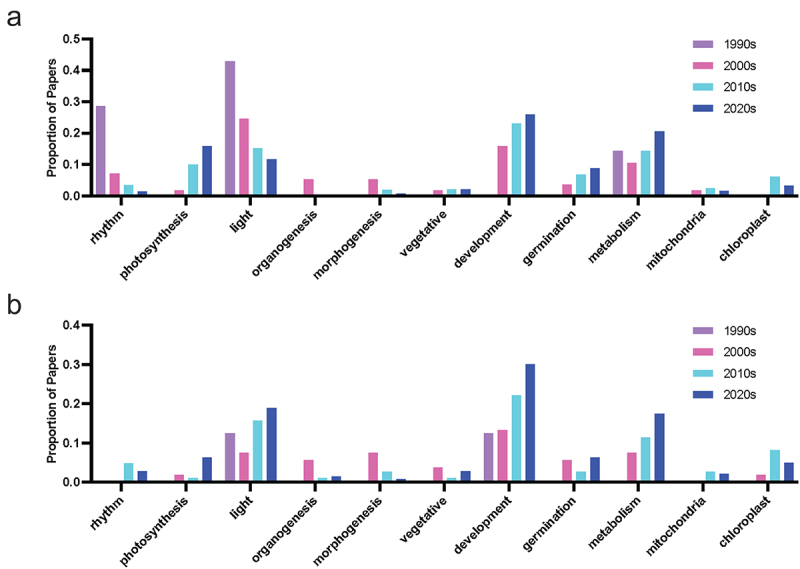
Proportion of publications per decade mentioning physiology-associated query terms in the abstract or title for (a) melatonin and (b) serotonin literature.

**Figure 9.**
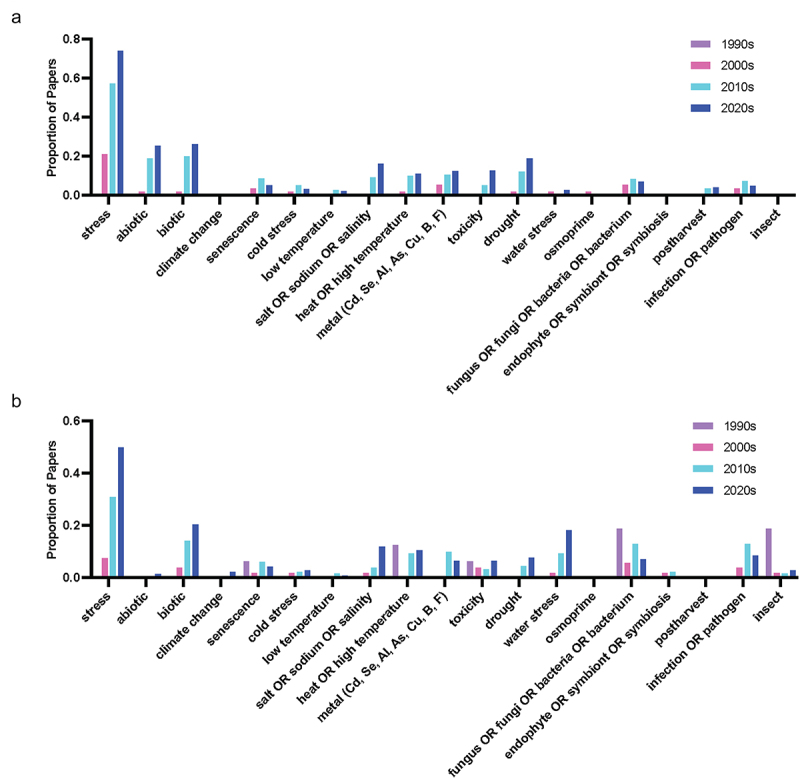
Proportion of publications per decade mentioning stress-associated query terms in the abstract or title for (a) melatonin and (b) serotonin literature.

Studies of morphogenetic functions and tissue culture experiments for both hormones have seen a decreasing trend in recent decades, with the use of terms including organogenesis, morphogenesis and tissue or *in vitro* culture reducing over time (Figure 9). The emphasis has shifted toward field-based studies of the stress-mitigating effects of both compounds (Figures 2 and 8), with an abundance of melatonin studies in plants documenting the capacity for melatonin to alleviate stress through both direct and indirect pathways. It will be interesting if more research is published on the physiological function of serotonin, if serotonin literature will follow a similar trend, or if this is reflective of the research focus of the different research groups publishing in these areas (Figure 3). Common effects of melatonin application include: improved redox status, upregulation of antioxidant pathways, improved photosynthetic efficiency, reduced chlorosis and tissue senescence. For example, up to 27% of serotonin papers, and 50% of melatonin papers mention the term ‘antioxidant’ in the title or abstract in the most recent decade. Surprisingly, there is a divergent trend between melatonin and serotonin, with respect to research that explicitly mentions the term light , with the proportion of research trending down for melatonin, and being relatively level for serotonin. Despite the trend though, about 10–20% of papers still include mention of the query term (Figure 8). Light is an interesting topic of research as it represents one of the classical mammalian effects of melatonin in particular as the chemical signal of darkness, well-defined interactions between melatonin, stress and blue light exposure in humans, and the regulation of circadian rhythms. This meant it was an area of active research early after melatonin’s discovery in plants, with some evidence of seasonal or daily rhythms linked to the wavelength of light or photoperiod.^46–48^ Early serotonin research in particular focused on the influence of light in the expression of the hormone in plants and suggested it may be a mediator of red-light exposure.^42,49,50^ Light, of course, is fundamental to plant life, driving photosynthesis, inducing abiotic stress when in excess (particularly in combination with other stress such as temperature or salinity) and serving as an important environmental signal. It is likely that new insights into indoleamine light signaling will follow.

Increasing adoption of molecular biology and -OMICs/multiomics approaches are also identifying an increasing suite of transcription factors that are influenced by melatonin and modulate downstream stress adaptations and responses these include among them (but not exhaustively): bZIP,^51^ MYB,^52^ MAPK,^53^ RBOH,^54^ COP1 and 9,^55^ CBF/DREB,^56^ ZAT,^57^ AUX/IAA,^58^ NAC,^59^ ERF^60^ and YABBY.^61^ Some excellent recent reviews detailing melatonin signaling pathways provide a comprehensive overview of these topics, and readers are referred to these for example.^5,62^ These, as well as other transcription factor families including WRKY,^58^ which has been found to regulate melatonin biosynthetic genes, are often associated with an improvement in reproductive capacity and/or yield in addition to, or as a result of, reduced stress. The primary emphasis of these studies is the identification of potential gene targets for use in breeding programs, particularly marker-assisted selection and gene-editing programs. A search does not find any current crops on the market that have been selected or edited specifically for their melatonin content, though transgenics have been used for decades to better understand melatonin function in plants.

Prior to 2020, no explicit mentions of climate change are included; however, since 2020, this area of focus is increasing (Figure 9). This aligns with both global funding priorities,^63^ and a general uptake in the framing of scientific research in the context of global climate change, as well as the need to identify rapid and inexpensive solutions to increasingly frequent extreme weather events for agriculture.^64^ Climate change threatens an estimated 20% of the world’s plant diversity^65^ and will present new challenges in the cultivation, harvest and management of commercial and wild crop species, as well as ecosystems. In the face of external challenges, plants cannot relocate, instead, they must rely on chemical defenses, which enable them to perceive environmental changes and respond by redirecting growth, detoxifying stress metabolites and stabilizing physiological processes. Recent literature has proposed that vulnerability is determined by three key factors: (a) exposure, i.e. extrinsic factors, (b) sensitivity, the degree to which the plant is affected by extrinsic factors, and (c) adaptive capacity, the ability for a plant to respond *in situ* to a changing climate and persist.^66,67^ Though the first two factors have been widely investigated, more research is still needed on the traits mediating adaptive capacity. Phytohormones are integral in mediating plant growth processes and are ideal mediators of plant plasticity and adaptive capacity due to their diversity and function as signaling molecules. Melatonin and serotonin, in particular, are excellent candidates for mediators of these responses due to their dual function in both stress mediation and modulation of plant growth outcomes.^29^ As the field of plant indoleamine research continues to expand the question of ‘how can a better understanding of endogenous indoleamines help us understand, conserve and preserve ecosystems and food systems?’, represents an exciting new frontier in plant indoleamine research. Answers to this question may start to be found in current trends in plant stress research. To answer it fully, however, researchers will need to draw on, and expand past areas of focus including fundamental studies of how plants grow, asking how plants make decisions on morphogenetic outcomes in response to these applied stresses, and what role plant hormones, in this discussion indoleamines, play at the molecular or cellular level. This in some ways brings the field full circle from its origins. For example, the first inference of the existence of hormones and particularly auxin in plants by Darwin in the Power of Movement in Plants used movement in response to environmental stimuli, particularly phototropism, to hypothesize the presence of a mobile growth regulation substance, which later was discovered to be auxin.^68^

## 3. Open Question: Interkingdom roles and interactions

**Figure 10.**
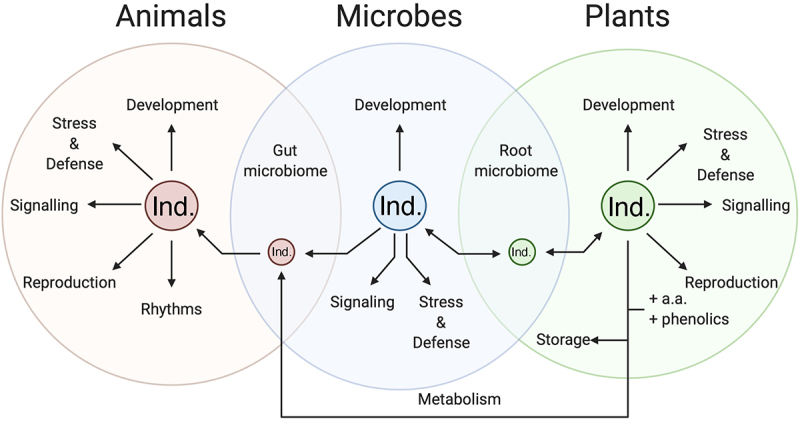
Summary of the interactions between and functions of serotonin in animal, microbial and plant cells and their associated microbiomes.

Melatonin and serotonin are increasingly being identified as important communication molecules not just within cells or individuals, but between individuals and species^69^ (Figure 10). One of the earliest and most robust areas of interaction is between serotonin and insects. Both melatonin and serotonin have been found to be induced in response to insect feeding.^70^ This is a clear stress response on the part of the plant to trigger downstream defensive pathways, while there is some research that suggests that it may also lead to effects on the insects themselves. For example, studies have shown that serotonin induces diverse signaling pathways in the Malpighian tubules of plant- feeding insects, including cAMP, and catecholamines.^71^ While this has primarily been studied in the context of serotonin secretion by the insects themselves, the Malpighian tubules are the gut of the insect and would therefore be exposed to plant-derived serotonin that is ingested, where it may play a role in modifying feeding behavior.^72^ Another emerging area of importance in this interkingdom communication is in the context of the microbiome.^73^ Recent papers on the human biome have led to discoveries of the importance of serotonin and dietary serotonin in mediating the gut−brain axis.^74^ The root system of plants serves as both the brain and the gut of the plant taking up nutrients and water and exploring the environment,^75^ and a model has been proposed that the gut−brain axis is an evolutionary advancement of the root−leaf axis in plants.^76^ The recent acknowledgment of the presence of the plant root microbiome makes this a particularly interesting new location for the discovery of plant indoleamine microbiome studies.^77^ The presence of an analogous system has been proposed; though the authors suggest that auxin may serve the function of serotonin in plants, it is likely coordination between indoleamines and auxin exists.^76^ Some research into melatonin in root microbe interactions has shown that associations with root symbionts can increase melatonin levels, while pathogenic bacterial infection has been found to induce a spike in both melatonin and serotonin levels.^78^ Mycorrhizal fungi are well understood to transfer organic nitrogen in the form of diverse protein and non-protein amino acids to plant cells providing proof of concept for the possibility of the tryptophan-derived serotonin transfer.^79^ The use of melatonin as a signaling molecule between root symbionts and grape vines has also been demonstrated, though the functions of this transfer have yet to be investigated.^80^ Melatonin treatment has also been found to promote the formation of these mycorrhizal symbioses.^81^ Plants have been shown to be able to undergo transport of indoleamines from the roots to the shoots, allowing for the establishment of hormone gradients reminiscent of auxin. The transport and localization patterns of serotonin have additionally recently been found to mimic that of auxin providing diverse pieces of a puzzle that has yet to be assembled.^29^ In tomatoes when the rootstock is grafted, a significant decrease in serotonin was observed, irrespective of the cultivar suggesting an important root−leaf dynamic exists that is interrupted in the grafting process though the mechanisms of this function are unclear.^82^

The presence of multidisciplinary studies and collaborations between plant scientists and human/medical researchers is likely to lead to further insights and should be a path forward including insights and applications into both systems. One example is the capacity of the human microbiome, through metabolism, to increase the bioavailability and bioactivity of conjugated or inert forms of hormones to more bioactive forms, e.g. through glycosylation/deglycosylation.^83^ We recently hypothesized that serotonin is present in diverse conjugated forms in addition to the established feruloyl-, sinapoyl-, caffeoyl- and cinnamoyl-phenolic conjugates.^84^ This may be leading to a dramatic underestimation of dietary serotonin availability. Paired with the recent discovery of the importance of dietary serotonin through interaction with the gut−brain axis,^85^ this may help us to understand the physiological impacts of specialized diets such as the Mediterranean diet which has been hypothesized to be partially effective due to the inclusion of high melatonin content foods.^86^

## 4. Open Question: Search for the phytoserotonin receptor

A phytomelatonin receptor, PMTR1, has now been identified.^87,88^ Despite initial concerns about the validity of this protein as a receptor vs a melatonin-interacting protein,^89^ it has now been studied in both monocots and dicots including maize,^90^ *Arabidopsis*^87,88^ and cassava.^91^ The primary downstream function identified for the receptor to date has been stomatal closure, associated with improved stress tolerance to osmotic and heat stress which has been found to occur via induction of nitric oxide signaling.^90,92,93^ Given the diversity of downstream effects melatonin has in plants, it is unlikely that this is the sole melatonin receptor in plants. A phytoserotonin receptor has remained more elusive. Seven classes of serotonin receptors have been reported: 5-HT_1–7_, all of which are ligand-gated ion channel G-coupled receptors. Over the years, research investigating the occurrence of serotonin receptors in non-mammalian organisms has led researchers to the hypothesis that serotonin receptor-mediated signaling arose early on during evolution. Studies involving known antagonists^36^ or labels suggest that serotonin receptor-mediated signaling in plants is plausible. Serotonin localization has been found to appear strikingly similar to that of auxin,^29^ with concentrations being highest in vascular tissues and supporting tissues particularly the pericycle, protophloem and protoxylem, and endodermis.^29,94,95^ This in conjunction with studies employing auxin receptors has led to the hypothesis that serotonin may interact with auxin receptors and transporters. This is supported by molecular studies which have suggested interaction with PIN2.^96^

Roshchina (2006) hypothesized that serotonin may interact with G-protein-coupled receptors at the cell’s surface, which in turn causes a conformational change to ion channel subunits. These events then lead to the modulation of cytoskeleton elements via actin and tubulin binding, as well as downstream effects within specific organelles through either mechanical or electrical waves.^97^ This was proposed based on studies on microspore development and pollen germination in knight’s star (*Hippeastrum* spp.) where serotonin was found to promote pollen germination in a cAMP-dependent manner.^98^ Similarly, both knight’s star and horsetail (*Equisetum arvense* L.) application of anti-contractile agents that disrupt microtubulin formation also inhibited the stimulatory effects from serotonin during microspore development.^99^

Another interesting aspect of serotonin and indoleamines generally is the presence of a strong chromophore in the aromatic ring structure of the molecule which is able to directly absorb light in the blue and UV wavelength range. Repeated tryptophan residues are used as photoreceptors in the UVR8 UV-B photoreceptors, where they directly absorb this light energy inducing conformational changes in the protein leading to translocation and downstream responses.^100,101^ Serotonin has been found to be responsive to blue light treatment in humans and plants, though the process is not well described in plants.^101,102^ Therefore, it is also possible that serotonin itself may be directly absorbing these wavelengths of light leading to disruptions in signaling or induction in stress mitigation cascades.

## 5. Open Question: Are melatonin and serotonin ‘safe’ and effective agrochemicals?

**Figure 11.**
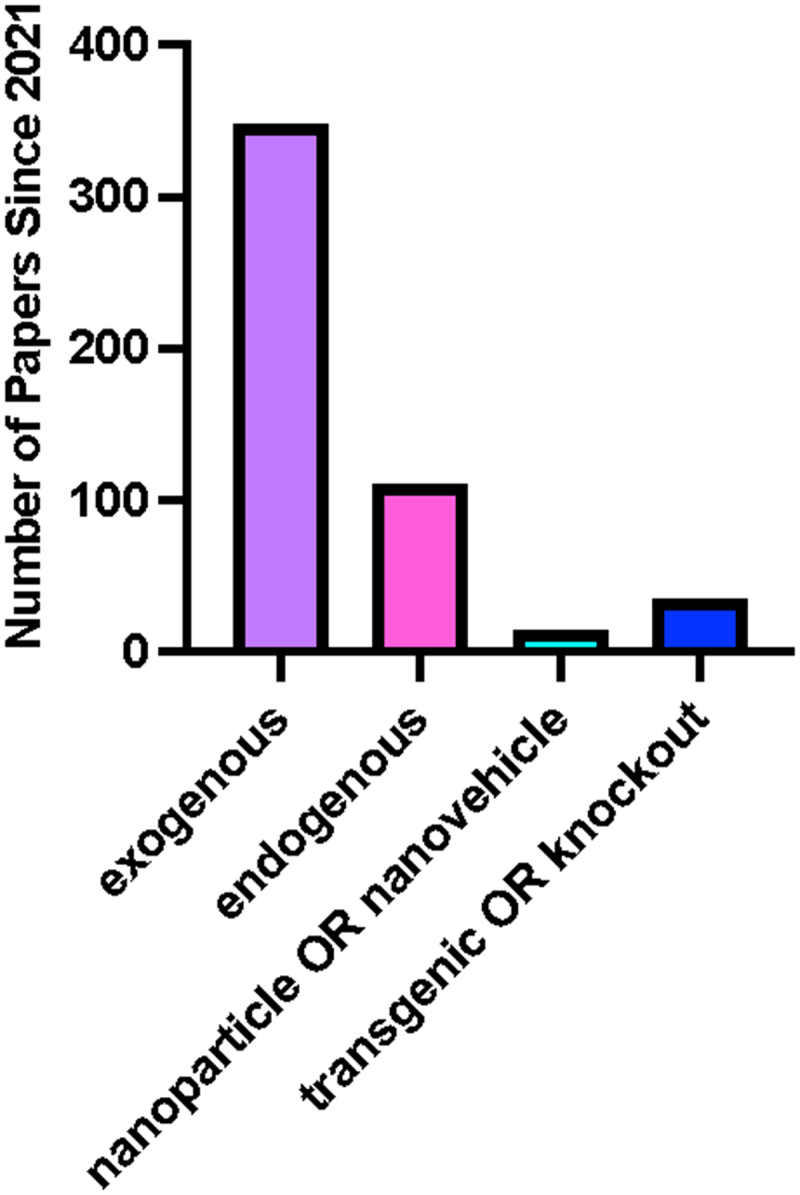
Overview of application methods of melatonin across all studies.

Despite rapidly increasing numbers of studies promoting exogenous application of melatonin in field settings as a potential solution to address increasingly challenging agricultural cultivation conditions, very little has been considered regarding the health and safety implications of this broad use to humans, wildlife and environments. Its presence, degradation and effects on the local flora and fauna of the targeted area are also not well documented. Melatonin is bioavailable to humans, and the potential impacts on human health, if it is persistent in environments, can be clearly traced thanks to decades of research in this area,^103,104^ while microbial communities in agricultural soils have been found to be modified in response to melatonin application.^105^ The limited data available highlight a deficit in policy and decision-making processes around melatonin use in agriculture.^106,107^ In Canada, pesticides are required to submit a list of findings that ’relates to the health or environmental risks or the value of the pest control product’ (Canadian Pesticide Act Section 7 Subsection 12); however, melatonin as a product may fall into the ‘bio-stimulant’ category which allows for leniency in what information needs to be made available, outside guaranteed analysis. With the gaps in our current understanding of melatonin and how its rate of application impacts the results or perceived effect of the treatment, displaying a labeled rate as a means to meet the regulations imposed by the Canadian Pesticide Act and/or the Canadian Fertilizer & Supplement Act is not achievable. High variability in application rates of melatonin represents an additional complexity in understanding the potential off-target effects or risks of melatonin use in agricultural settings. Some new efforts are now examining alternate delivery mechanisms through the use of nano-vehicle, nanoparticle or nano-delivery systems requiring encapsulation of melatonin within nanoparticles (Figure 11).^108,109^ While these systems may lead to more effective uptake or efficient application, leading to reduced application rates, whether this will lead to a net improvement is still yet to be fully defined. Examples from other nanoparticles include the use of some metal-based (e.g. zinc and copper) nanoparticles in agricultural as well as other settings (e.g. zinc sunscreens) which is now leading to metal accumulation in agricultural soils; for example, in rice paddies where downstream effects of plant growth and metabolism are being observed.^110^ Compared to other application systems, this area is relatively underinvestigated and many of these questions are likely to be answered as more knowledge is generated in the literature (Figure 10). Given the increasing use of hormones in plant agriculture settings and the existing uncertainty of the full impacts of long-term exposure to or the use of melatonin supplements developed for human consumption^111^ in the literature, a better understanding of the persistence and effects of melatonin as an agricultural product should be a priority area for consideration.

## Supplementary Material

Supplemental Material

## Data Availability

All data referenced in the manuscript may be accessed at: DOI 10.17605/OSF.IO/N3K8U.
