# Supplementary material for: Views and perspectives on the indoleamines serotonin and melatonin in plants: past, present and future
Source: Plant Signal Behav. 2024 Jun 20;19(1):2366545. doi: 10.1080/15592324.2024.2366545 (PMC11195476; doi:10.1080/15592324.2024.2366545)
Supplement: Supplementary file 1 [file KPSB_A_2366545_SM9982.odt]

Supplementary Materials

Supplementary File 1. List of all retrieved records for melatonin AND plants organized by topic query

Supplementary File 2. List of all retrieved records for serotonin AND plants organized by topic query

Supplementary File 3. List of all retrieved records for melatonin AND plants organized by species query

Supplementary File 4. List of all retrieved records for serotonin AND plants organized by species query

Supplementary File 5. Trends in plant melatonin research by decade from 1990-2024 both by total number of published paper, and proportion of published papers. Sparklines visualize trends, while slope is calculated by each of the first year of the decade (e.g. 1990, 2000, 2010, 2020).

Supplementary File 6. Trends in plant serotonin research by decade from 1990-2024 both by total number of published paper, and proportion of published papers. Sparklines visualize trends, while slope is calculated by each of the first year of the decade (e.g. 1990, 2000, 2010, 2020).
